# Profiling of Serum Metabolites of Acute Intermittent Porphyria and Asymptomatic HMBS Mutation Carriers

**DOI:** 10.3390/cells10102579

**Published:** 2021-09-28

**Authors:** Chia-Ni Lin, Ming-Shi Shiao, Mei-Ling Cheng, Chiung-Mei Chen, Hung-Chou Kuo

**Affiliations:** 1 Department of Laboratory Medicine, Chang Gung Memorial Hospital, Taoyuan 333, Taiwan; chianilin@cgmh.org.tw; 2 Department of Medical Biotechnology and Laboratory Science, College of Medicine, Chang Gung University, Taoyuan 333, Taiwan; 3 Metabolomics Core Laboratory, Healthy Aging Research Center, Chang Gung University, Taoyuan 333, Taiwan; msshiao@mail.cgu.edu.tw (M.-S.S.); chengm@mail.cgu.edu.tw (M.-L.C.); 4 Department of Biomedical Sciences, College of Medicine, Chang Gung University, Taoyuan 333, Taiwan; 5 Graduate Institute of Biomedical Sciences, College of Medicine, Chang Gung University, Taoyuan 333, Taiwan; 6 Clinical Metabolomics Core Laboratory, Chang Gung Memorial Hospital at Linkou, Taoyuan 333, Taiwan; 7 Linkou Medical Center, Department of Neurology, Chang Gung Memorial Hospital, College of Medicine, Chang Gung University, Taoyuan 333, Taiwan; cmchen@adm.cgmh.org.tw

**Keywords:** porphobilinogen deaminase, metabolomic profiling, metabolic reprogramming, heme metabolism, δ-aminolevulinic acid, porphobilinogen

## Abstract

This study aims to present the serum metabolite profiles of patients with acute intermittent porphyria (AIP) and identify specific metabolites that could potentially discriminate between AIP, asymptomatic HMBS mutation carriers, and healthy individuals. The study cohort included 46 female participants: 21 AIP patients, 5 asymptomatic carriers, and 20 ‘normal’ participants (without HMBS gene mutation). Serum samples were analyzed for 157 selected metabolites or clinical variables using an assay combining liquid chromatography MS/MS and direct flow injection. AUC analysis was used to distinguish unique variables between the three groups. A total of 15 variables differed significantly between the AIP and normal control group (VIP score > 1.0 and *p* < 0.05 with FDR correction). In AIP patients, the levels tyrosine, valine, and eGFR were significantly lower, and the levels of sphingomyelin C16:0, C24:0, C24:1, phosphatidylcholine diacyl C32:1, C36:1, C36:3, ornithine, sarcosine, citrulline, blood urea nitrogen AST, and ALT were significantly higher. The AUC of these 15 variables in discriminating between normal and AIP patients ranged between 0.73 and 0.94 (*p* < 0.05). In conclusion, serum metabolic profiles differ between normal individuals and patients carrying the HMBS mutation. The unique metabolites associated with AIP identified in this study may be useful for monitoring the development of AIP symptoms.

## 1. Introduction

Acute intermittent porphyria (AIP) is an autosomal dominant disorder caused by deficient activity of porphobilinogen deaminase (PBGD), the third enzyme in the heme biosynthetic pathway (due to mutation in HMBS gene) [1,2]. In the absence of sufficient PBGD activity, the heme precursors porphobilinogen (PBG) and δ-aminolevulinic acid (ALA) accumulate in the body, promoting acute, potentially life-threatening neurovisceral attacks [3]. The clinical symptoms of AIP include abdominal pain, nausea, constipation, and increased heart rate and blood pressure. Some patients develop severe hyponatremia, limb weakness, peripheral motor nerve paralysis, neuralgia, and seizures, constituting a severe neurological emergency. For reasons unknown, some carriers of HMBS mutations have frequent attacks while others remain asymptomatic, and the severity of symptoms varies between patients [4]. Even though some individuals with an HMBS gene mutation are able to minimize AIP attacks by avoiding exposure to known predisposing risk factors [5], currently, there is yet no clear explanation as to why symptoms are absent in some HMBS gene mutation carriers. Therefore, monitoring for disease development in patients with an HMBS mutation is important to AIP prevention. The identification of biomarkers indicating a risk for symptom development would greatly facilitate the clinical management of these patients and shed light on the broader metabolic consequences of a deficiency in the PBGD enzyme.

The precise pathogenetic mechanism that triggers acute AIP attacks is unknown. Attacks are precipitated by a variety of factors, including reproductive hormones during the menstrual cycle, alcohol consumption, medications, weight loss, and infections [5]. These factors promote the attacks by inducing ALAS1 transcription or ALA synthase activity, either directly or indirectly [6], thereby increasing the accumulation of neurotoxic ALA and PBG. Growing evidence suggests that this block in the heme synthesis pathway results in metabolic changes that leave AIP patients vulnerable to recurrent attacks. A study using 1H-NMR to compare urinary metabolites between AIP patients with and without recurrent attacks identified elevated urinary glycine, a precursor to ALA, as unique to those patients with recurrence, indicating a disturbance in glycine metabolism that may underlie recurrence [7]. Urine 1H-NMR profiling revealed that asymptomatic HMBS gene carriers had altered levels of acetate, citrate, and pyruvate. Because these metabolites are involved in glycolysis and energy-conversion pathways, these findings suggest that metabolic reprograming occurs in AIP patients even in the absence of overt symptoms [8]. These studies indicate the potential of metabolome analysis to reveal the mechanism underlying the intermittent nature of AIP and identify markers predictive of AIP attack risk. Comprehensive studies of the plasma AIP metabolome have not been reported.

This study aims to determine whether serum metabolic profiles differ between AIP patients, asymptomatic carriers, and healthy individuals and to identify specific metabolites that could potentially discriminate between them. Alterations in the metabolomic profile may provide clues regarding the pathophysiologic processes underlying AIP disease progression.

## 2. Materials and Methods

### 2.1. Design, Setting, and Participants

The study cohort included 21 AIP patients, 5 asymptomatic carriers (with the HMBS gene mutation but asymptomatic), and 20 normal individuals (family members of the AIP patients and healthy volunteers not related to AIP patients) who were diagnosed and followed at the Chang Gung Memorial Hospital between 1 February 2019 and 31 January 2020. Only females were included in this study. The status of all patients, asymptomatic carriers, and healthy family members was confirmed by HMBS gene mutation analysis; PBGD activity in erythrocytes and urinary PBG and ALA measurements were also recorded for patients and asymptomatic carriers. Significantly elevated δ-ALA and PBG levels in 24 h urine were observed in AIP patients with acute attacks. All asymptomatic carriers had no medical history of abdominal pain.

Participants’ clinical data were collected, including body mass index, disease history (including diabetes, hypertension, heart failure, and other metabolic diseases), medication history, and lifestyle factors such as smoking and drinking. After a 12 h fasting, serum samples were collected from all participants for metabolite analysis. The serum sample for AIP patients was also collected at baseline, not during acute attack.

### 2.2. Metabolite Analysis

Serum metabolites were analyzed using the targeted Absolute IDQ^®®^p180 kit (Biocrates Life Science, AG, Innsbruck, Austria), which combines a liquid chromatography MS/MS assay and a direct flow injection assay. This assay allows for the identification and quantification of 185 endogenous metabolites from 5 different compound classes, including acylcarnitines, amino acids, biogenic amines, sugars, sphingomyelins, and glycerophospholipids. The assay was performed using a Waters Acquity Xevo TQ-S instrument (Waters, Milford, MA, USA) according to the manufacturer’s instructions. Briefly, the serum samples were thawed, vortexed, and centrifuged at 13,000× *g*. A 10-uL aliquot of the sample supernatant was loaded onto filter paper, dried under nitrogen flow, and derivatized by the addition of 20 µL of 5% phenyl-isothiocyanate for 20 min. The filter spots were dried under nitrogen flow for 45 min, and the metabolites were extracted by the addition of 300 µL of methanol containing 5 mM ammonium acetate. The extracts were then analyzed by mass spectrometry. The extracts were injected onto an Acquity UPLC BEH C18 (2.1 × 75 mm, 1.7-μm particle size, Waters, Milford, MA, USA) at 50 °C for chromatographic separation of amino acids and biogenic amines, operated in negative electrospray ionization and multiple reaction monitoring (MRM) mode, followed by FIA-MS/MS of sphingolipids, hexoses, acylcarnitines, and glycerolipids. Quantitation of LC data was performed with TargetLynx (Waters, Milford, MA, USA) based on an external 7-point calibration. FIA data were converted and imported into the Biocrates^®®^ MetIDQ™ software.

### 2.3. Statistical Analysis

Continuous variables were analyzed using Kruskal–Wallis test or Mann–Whitney U test, and the data are presented as the median and interquartile. Categorical variables were analyzed using the chi-square test or Fisher’s exact test, and the data are presented as counts and percentages. The clinical variables and metabolites were analyzed using principal components analysis (PCA) and partial least squares discriminant analysis (PLS-DA) through the web-based metabolomics software MetaboAnalyst 5.0. All metabolites were normalized by Pareto scaling. The variable importance in the projection (VIP) of each variable in the model was calculated to indicate its contribution to the classification. A higher VIP value indicates a stronger contribution to discrimination between groups. VIP values greater than 1.0 were considered significantly different. The area under the receiver operating characteristic curve (AUC) analysis was used to distinguish between AIP and non-AIP participants. Significance was set as two-sided *p* < 0.05 for Dunn’s post hoc test and false discovery rate (FDR) correction. All statistical analyses were performed using SAS version 9.4, Windows NT version (SAS Institute, Inc., Cary, NC, USA).

## 3. Results

### 3.1. Participant Characteristics

The study cohort included a total of 46 participants: 21 AIP patients, 5 with HMBS gene mutation, and 20 normal subjects. The phenotypical characteristics, including initial phenotype, genotype, severity, chronic symptoms, ALA and PBG measurements of the asymptomatic carriers and AIP patients, are shown in Supplement Table S1. Baseline characteristics of the non-AIP and AIP groups are shown in Table 1. Blood urea nitrogen (BUN), aspartate aminotransferase (AST), alanine aminotransferase (ALT), total cholesterol (T-CHOL), high-density lipoprotein cholesterol (HDL-C), and creatinine were significantly higher in the AIP group (all *p* < 0.05). The estimated glomerular filtration rate (eGFR) was significantly lower in the AIP group (*p* < 0.0001) (Table 1). The distribution of 144 metabolites for normal, asymptomatic carriers, and AIP groups are shown in Table S1.

### 3.2. Comparison of Clinical Variables and Metabolite Levels between Normal, Asymptomatic Carriers, and AIP Patients

**Figure 1:**
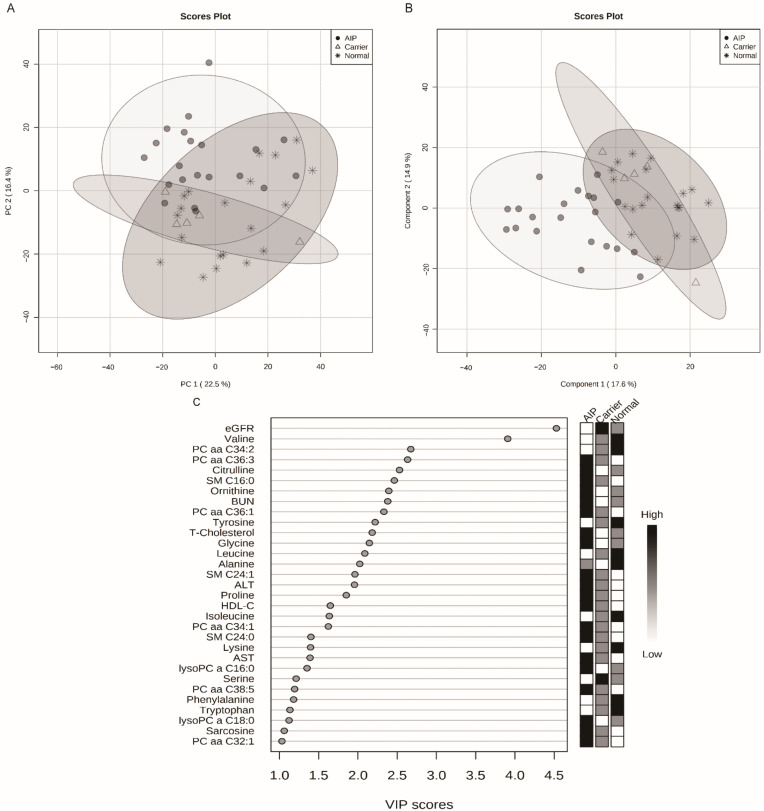
Targeted metabolomics profiles of normal, asymptomatic carriers, and AIP patients. Extracted plasma from AIP patients (n = 21), asymptomatic carriers (n = 5), and normal (n = 20) were analyzed by LC–MS/MS and direct flow injection assay. (**A**) Principal component analysis (PCA) did not demonstrate a clear separation between the three groups. (**B**) The partial least squares discriminant analysis (PLS-DA) demonstrates a clear separation of variables between groups with and without AIP (R2Y = 0.54, Q2 = 0.38). (**C**) Metabolites and clinical variables with variable importance in the projection (VIP) score > 1.0, indicating their contribution to the classification in the PLS-DA model. eGFR—estimated glomerular filtration rate; R2Y—cumulative variation in the Y matrix; Q2—predictive performance of the model.

PCA and PLS-DA results for 13 clinical variables (age, BMI, BUN, AST, ALT, AC, Hb-Alc, T-CHOL, HDL-C, LDL-C, triglyceride, creatinine, and eGFR) and 144 metabolites between the normal, asymptomatic carriers, and AIP patients are shown in Figure 1. The PCA results showed that there was no clear separation between normal, asymptomatic carriers and AIP patients (Figure 1A). However, the PLS-DA model clearly separated the normal, asymptomatic carriers, and AIP patient groups (R2, 0.54; Q2, 0.38) (Figure 1B). Metabolites and clinical variables with a VIP score > 1.0 are listed in Figure 1C.

**Figure 2:**
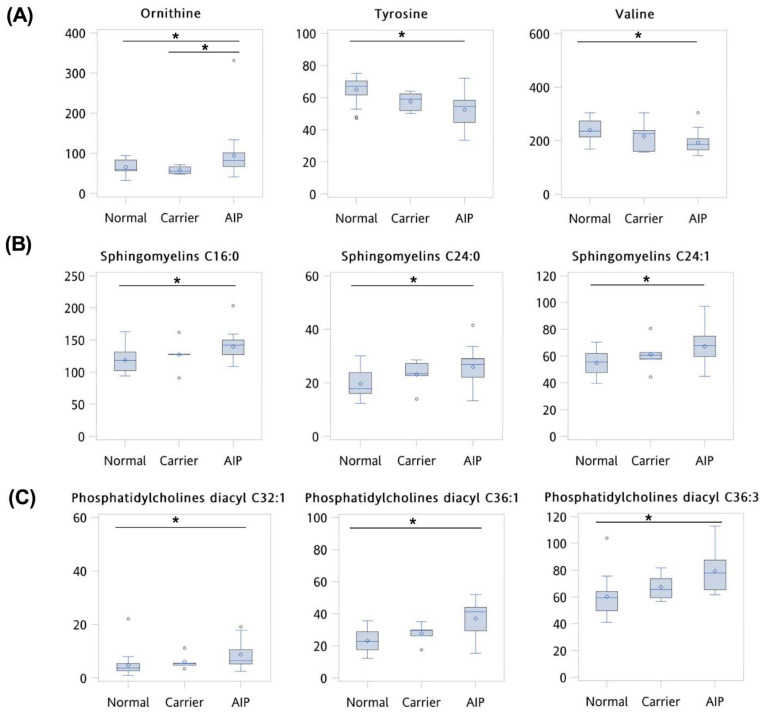

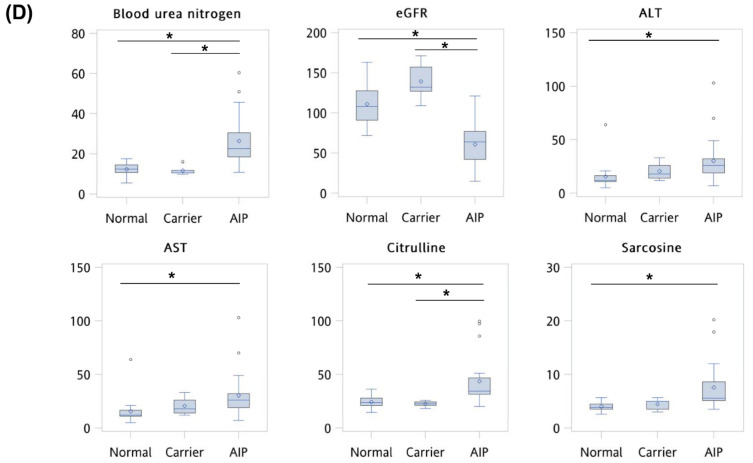
Clinical variables and metabolite levels differed significantly between normal, asymptomatic carriers, and AIP patients. Statistical differences were determined by one-way ANOVA, and data are presented as minimum, first quartile, third quartile, and maximum (line, median value; diamond, mean value; circle, outlier value) in box-and-whisker plots. Pair-wise comparison was executed using Dunn’s post hoc test. (**A**) Amino acids; (**B**) Sphingomyelins; (**C**) Glycerophospholipids; (**D**) Kidney function, urea cycle, and other serum markers. * *p* < 0.05 for Dunn’s post hoc test.

A significant difference was observed in 15 metabolites or clinical variables between normal, asymptomatic carriers, and AIP patients (VIP score > 1.0 and *p* < 0.05 of FDR correction) (Figure 2). Compared to the normal and asymptomatic carrier groups, the AIP group exhibiting significantly lower levels of amino acid tyrosine (median: 67.1, 59.1, and 54.4 µM, respectively) and valine (median: 235.5, 227.0, and 186.0 µM, respectively) and lower eGFR (mean: 108.0, 132.0, and 64.0 mL/min/1.73 m^2^, respectively).

Compared to the normal and asymptomatic carrier groups, patients in the AIP group had significantly higher levels of ornithine (median: 60.3, 56.1, and 82.0 µM, respectively), sphingomyelin C16:0 (median: 118.5, 127.0, and 142.0 µM, respectively), sphingomyelin C24:0 (median: 17.8, 23.4, and 26.9 µM, respectively), sphingomyelin C24:1 (median: 55.8, 60.7, and 67.8 µM, respectively), phosphatidylcholine diacyl C32:1 (median: 3.8, 5.5, and 6.6 µM, respectively), phosphatidylcholine diacyl C36:1 (median: 22.7, 29.6, and 41.2 µM, respectively), phosphatidylcholine diacyl C36:3 (median: 59.3, 65.5, and 77.8 µM, respectively), BUN (median: 12.5, 10.5, and 22.6, respectively), AST (median: 21.0, 23.0, and 28.0 µM, respectively), ALT (median: 12.0, 18.0, and 26.0 µM, respectively), citrulline (median: 23.9, 22.8, and 34.3 µM, respectively), and sarcosine (median: 3.9, 5.0, and 5.6 mg/dL, respectively) (Figure 2).

**Figure 3:**
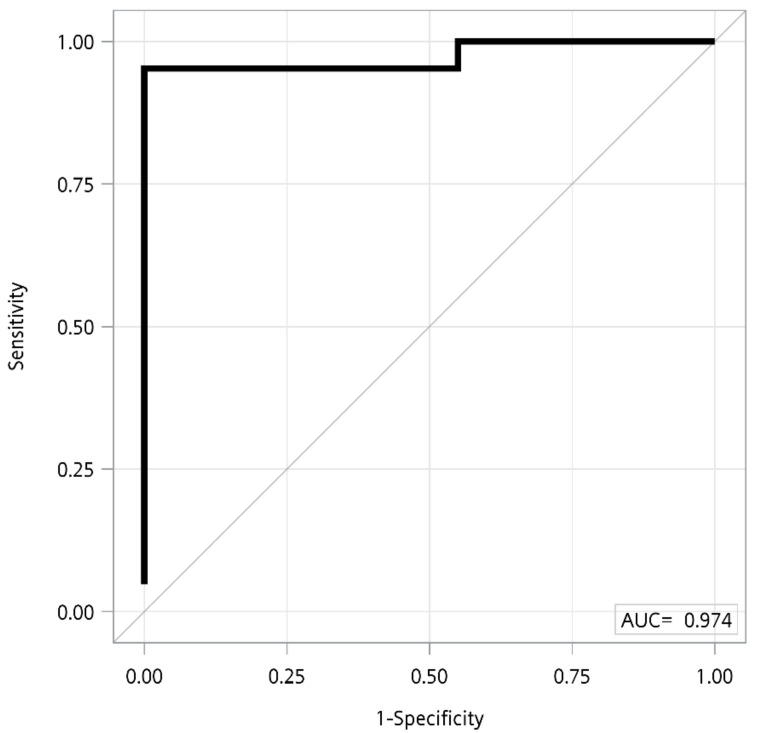
Area under the curve analysis of selected variables for determining AIP. AUC between control and AIP, 0.97; model includes eGFR and sphingomyelin C24:0.

After selecting for differences in metabolites or clinical variables between normal, asymptomatic carriers, and AIP patients by VIP score > 1.0 and *p* < 0.05 of FDR correction, the results of AUC analysis of 15 selected variables for discriminating between normal, asymptomatic carriers, and AIP patients are shown in Table 2. No marker was identified for discriminating between normal and asymptomatic carriers nor between asymptomatic carriers and AIP patients after FDR correction. For discriminating between normal and AIP patients, 15 variables were differed significantly and had FDR < 0.05: ornithine, tyrosine, valine, sphingomyelin C16:0, C24:0, C24:1, phosphatidylcholine diacyl C32:1, C36:1, C36:3, BUN, eGFR, ALT, AST, citrulline, and sarcosine (AUC range, 0.73–0.94) (Table 2). Step-wise selection of variables showed that the combination of eGFR and sphingomyelin C24:0 could discriminate between AIP and normal individuals with 0.97 AUC (Figure 3).

## 4. Discussion

In this metabolomic study of serum acylcarnitines, amino acids, biogenic amines, sugars, sphingomyelins, and glycerophospholipids in AIP patients, we identified 15 variables that differed between normal and AIP patients at baseline (not during an acute attack). The levels of amino acid tyrosine, valine, and eGFR were significantly lower in AIP patients. The levels of sphingomyelin C16:0, C24:0, C24:1, phosphatidylcholine diacyl C32:1, C36:1, C36:3, BUN, citrulline, ornithine, and sarcosine were elevated in AIP patients. The AUC of these 15 variables was high (range, 0.74–0.94). These findings indicate clear differences in the serum metabolic profiles between AIP patients and normal individuals.

The study of metabolites using the recently developed metabolomic approach has revealed disruptions in metabolism associated with a variety of human diseases, providing clues to their underlying pathogenetic mechanisms and biomarkers of disease identity and severity. Such studies have identified plasma amino acids and lipid species associated with abnormal glucose metabolism and insulin resistance [9], biomarkers for the progression of hepatitis to hepatocellular carcinoma [10], and metabolic profiles indicative of diabetic kidney disease [11]. Studies of urine metabolites in AIP have revealed a number of disease markers, including metabolites involved in glycolysis and energy-conversion pathways such as acetate, citrate, and pyruvate [8], and steroid 5α-reductase metabolites [12]. Our results contribute to this growing body of metabolome knowledge, identifying specific serum metabolites that are altered in AIP.

**Figure 4:**
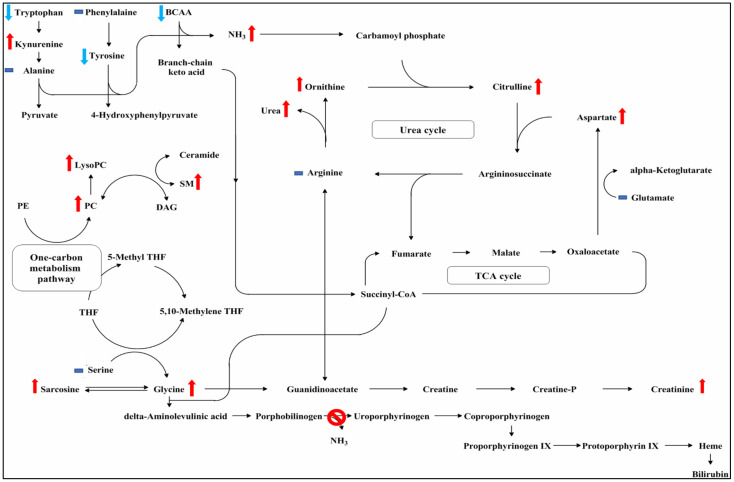
Putative metabolic pathway based on serum metabolic profiling results. Baseline serum metabolic profiles differ between normal participants and AIP patients, including amino acids, sphingomyelins, glycerophospholipids, and markers for urea cycle, TCA cycle, glycine, and creatinine synthesis. In AIP patients, the levels of branched-chain amino acids (BCAA: Valine, Leucine, Isoleucine), and aromatic amino acids (AAA: Tryptophan, Tyrosine), were significantly lower (indicated by light blue arrows); levels of the sphingomyelin (SM) (C16:0, C24:0, C24:1), phosphatidylcholine diacyl (C32:1, C36:1, C36:3), and LysoPC in the one-carbon metabolism pathway were higher (indicated by red arrows). Alterations in glycine synthesis, creatinine metabolism, and urea cycle were evident from the elevated levels of serine, sarcosine, glycine, urea, citrulline, ornithine, and aspartate in AIP patients (indicated by red arrows). Blue horizontal bar indicates no difference. Unlabeled metabolites were not quantified in this study.

In addition to the 15 discriminating variables between AIP and normal control, metabolites with altered expression levels between the three groups which fall outside the defined range of statistical significance (i.e., statistically significant but did not fulfil the criteria of VIP score > 1.0 and *p* < 0.05 of FDR correction) were examined. It was found that the level of kynurenine, glycine, aspartate, LysoPC, and PCs were higher for AIP patients, whereas branched-chain amino acids (BCAA: Valine, Leucine, Isoleucine), aromatic amino acids (AAA: Tryptophan, Tyrosine) were lower for AIP patients (Supplement Table S2). Using the serum metabolic profile results obtained in this study, a putative AIP metabolic pathway was proposed (Figure 4). Note that considering that AIP patients may have problems with renal function (as demonstrated by lower eGFR value the AIP patient), it was likely that some of the differences in clinical variables or metabolites concentrations were related to altered renal function, e.g., elevated BUN and creatinine.

Defects in synthesizing Heme (problem with conversion of porphobilinogen to uroporphyrinogen) could have resulted in accumulation of glycine and sarcosine, which is an intermediate and byproduct in glycine synthesis (glycine, sarcosine). Alteration in glycine synthesis could affect: 1. its catabolism (via serine) into pyruvate, consequently affecting entering of TCA cycle; 2. one-carbon metabolism pathway (LysoPC, PC, SM); 3. the concentration of guanidinoacetate in creatinine metabolism. Elevated levels of guanidinoacetate may exert neurotoxic effects. Creatinine metabolism is important to urea cycle regulation, alteration to creatine metabolism subsequently affects arginine and consequently affects the urea cycle (citrulline, aspartate). Additionally, a low level of both BCAA and aromatic amino acids may have affected the synthesis of carbamoyl phosphate, which is normally converted to citrulline when condensed with ornithine during the urea cycle. In urea synthesis, citrulline condenses with aspartate to produce argininosuccinate, which cleaved to arginine, releasing urea when hydrolyzed by arginase. In AIP patients, decreased BCAA and AAA levels may have resulted in defects in the urea cycle (represented by the altered levels of NH3, citrulline, and aspartate, and creatinine metabolism (represented by altered levels of sarcosine and glycine). The putative metabolic pathway based on our serum profiling results is shown in Figure 4.

We observed that asymptomatic carriers and AIP patients differ metabolically, as the set of variables that distinguish normal from AIP patients differed from that distinguish asymptomatic carriers from AIP patients. The most striking differences between normal and AIP patient groups were in the levels of branched-chain amino acids, aromatic amino acids, glycine, sphingomyelins, and phosphatidylcholines. As the catabolic end products of a multitude of biochemical pathways and precursors to important biomolecules, serum amino acids can serve as powerful indicators of metabolic disruption. The branched-chain amino acids (valine, leucine, and isoleucine) are substrates for protein synthesis and energy production and perform metabolic and signaling functions [13]. Consistent with previous studies of urine metabolites [7,8], we observed lower levels of all three branched-chain amino acids in the serum of AIP patients. While the mechanism underlying this observation is unclear, decreased BCAAs have been reported in liver cirrhosis, urea cycle disorders, and chronic renal insufficiency. Low serum BCAA levels play a role in the pathogenesis of hepatic encephalopathy and muscle wasting in liver cirrhosis [14,15], and renal dysfunction can also affect BCAA metabolism [16]. Increased metabolic consumption of BCAA to detoxify ammonia to glutamine in muscles is the cause of decreased BCAA levels in liver cirrhosis and urea cycle disorders, while increased branched-chain keto acid dehydrogenase activity causes elevated levels of BCAA oxidation in chronic renal failure, trauma, burn, sepsis, cancer, phenylbutyrate-treated subjects, and during exercise [13].

We also observed lower serum levels of all the aromatic amino acids (tyrosine and tryptophan) in AIP patients. Aromatic amino acids serve as precursors for essential neurotransmitters and hormones. Our finding is supported by previous reports of altered tryptophan metabolism in AIP. Altered tryptophan metabolism has been implicated in the neurological manifestations of acute porphyria [17]. Compared to normal controls, AIP patients were shown to have significantly modified ratios of tryptophan catabolites in urine that suggested alterations in the activity of two enzymes, leading to elevated kynurenine levels [18]. Decreased nocturnal levels of plasma melatonin, produced from tryptophan via serotonin, were observed in AIP women [19].

AIP patients are at higher risk for liver cirrhosis and hepatocellular carcinoma [20] and chronic kidney disease [21]. Several of the metabolic differences we identified as distinguishing AIP patients are related to liver and kidney function (i.e., creatinine and BUN). The neurological damage in AIP is caused by the accumulation of PBG and ALA, the reaction product of glycine and succinyl-CoA [22]. We observed that glycine, synthesized primarily in the liver and kidneys [23], was significantly elevated in the serum of AIP patients, compared to normal. A study of urine metabolites in AIP patients with frequent recurrence revealed elevated glycine concentrations at the end of treatment, indicating that glycine metabolic reprogramming occurs in these patients and is associated with recurrence [7]. As the liver is the primary site of lipid synthesis, our finding that several sphingomyelins and phosphatidylcholines were elevated in AIP suggests alterations in liver metabolism in these patients. Phosphatidylcholine diacyl C36:3, elevated in our AIP patients, was previously identified as a promising predictive biomarker of hepatic lipidosis in dairy cattle [24], indicating altered lipid metabolism in the liver. Differences in kidney function between the patient groups are suggested by our AUC analysis, which revealed a strikingly high sensitivity of the combined markers eGFR + sphingomyelin C24:0 to distinguish between normal and AIP patients (AUC, 0.97).

Metabolites that distinguish between AIP patients and asymptomatic carries were not identified in this study. AIP manifestation is very complicated and is likely affected by other factors not studied in this study; differences in other genes or genetic factors can be the cause of the difference in metabolites between asymptomatic carriers and patients. Molecular genetic studies using whole-exome sequencing have shown that mutations carriers of both the HMBS gene and the ALAD gene may not necessarily affect AIP clinical manifestation, and mutations in genes regulating nervous system genes contributed to AIP manifestation [25]. Studies investigating possible contribution of mutations in genes regulating AIP manifestation and metabolites is warranted; however, they would require a larger sample of AIP patients and their asymptomatic relatives.

This study has several limitations. The normal control group include family members of AIP patients and thus would share same genetic background with AIP patients and asymptomatic HMBS mutation carriers. The patient cohort was not separated according to specific HMBS mutation or level of enzyme activity, so the influence of genetic variants on the results cannot be excluded. Such distinction would be difficult because the HMBS mutations in AIP are widely heterogeneous, with many mutations limited to a single or small group of families [4]. Future studies using ratios of metabolite concentrations may also provide additional information, as this method can substitute for enzyme concentrations for pairs of metabolites closely connected to the direct substrates and products of a given enzymatic reaction [26].

## 5. Conclusions

This study presented the serum metabolic profiles of symptomatic AIP patients, asymptomatic carriers of HMBS mutations, and normal controls. The variables observed to be significantly associated with AIP include multiple amino acid precursors to neurotransmitters and the heme precursor glycine. A unique finding was the strong correlation between AIP and the combination of eGFR and sphingomyelin. The metabolic differences between AIP patients, asymptomatic carriers, and normal control may provide clues to elucidating the physiologic processes underlying AIP disease progression.

## Figures and Tables

**Table 1:** Baseline characteristics of normal, asymptomatic carriers, and AIP patients.

	Normal	Carrier	AIP	*p*-Value
	N = 20	N = 5	N = 21	
Age (y)	41 (34.50, 48.00)	42 (29.00, 45.00)	43 (34.00, 51.00)	0.74
BMI (kg/m^2^)	21.90 (20.50, 26.80)	24.10 (22.90, 24.40)	22.50 (19.50, 24.80)	0.76
BUN (mg/dL)	12.45 (10.75, 14.60)	10.50 (10.50, 11.80)	22.60 (18.40, 30.50) ^‡^^§^	<0.0001 *
AST (U/L)	21.00 (19.00, 22.00)	23.00 (22.00, 25.00)	28.00 (25.00, 30.00) ^‡^	0.0003 *
ALT (U/L)	12.00 (11.00, 16.50)	18.00 (14.00, 26.00)	26.00 (19.00, 32.00) ^‡^	0.001 *
AC (mg/dL)	86.50 (81.50, 96.50)	90.00 (83.00, 90.00)	88.00 (82.00, 91.00)	0.98
Hb-Alc (%)	5.40 (5.20, 5.50)	5.20 (5.10, 5.50)	5.40 (5.00, 5.60)	0.99
T-CHOL (mg/dL)	183.50 (156.00, 196.50)	169.00 (161.00, 172.00)	200.00 (184.00, 217.00)	0.04 *
HDL-C (mg/dL)	55.00 (48.50, 67.00)	66.00 (58.00, 75.00)	68.00 (60.00, 77.00) ^‡^	0.03 *
LDL-C (mg/dL)	101.50 (85.00, 118.00)	95.00 (85.00, 98.00)	104.00 (100.00, 117.00)	0.30
Triglyceride (mg/dL)	74.00 (63.00, 120.50)	51.00 (47.00, 58.00)	87.00 (68.00, 125.00)	0.12
Creatinine (mg/dL)	0.62 (0.54, 0.68)	0.51 (0.47, 0.52)	1.00 (0.80, 1.37) ^‡^^§^	<0.0001 *
eGFR (mL/min/1.73 m^2^)	108.00 (91.00, 127.50)	132.00 (127.00, 157.00)	64.00 (42.00, 77.00) ^‡^^§^	<0.0001 *

AIP—acute intermittent porphyria; BMI—body mass index; BUN—blood urea nitrogen; AST—aspartate aminotransferase; ALT—alanine aminotransferase; AC—fasting blood glucose; Hb-A1c—glycated hemoglobin; T-CHOL—total cholesterol; HDL-C—high-density lipoprotein cholesterol; LDL-C—low-density lipoprotein cholesterol; eGFR—estimated glomerular filtration rate. Data are presented as median and interquartile. * *p* < 0.05 by Kruskal–Wallis test. ^‡^ *p* < 0.05 for Dunn’s multiple comparison test between normal and AIP group. ^§^ *p* < 0.05 for Dunn’s multiple comparison test between carrier and AIP group.

**Table 2:** Variables identified by AUC analysis as distinguishing AIP patients from asymptomatic carriers and from normal participants.

Variables (µM)	AUC	*p*	FDR	Sensitivity	Specificity
Carrier vs. AIP					
Blood urea nitrogen	0.962	**0.026**	0.233	0.857	1.000
Citrulline	0.910	**0.049**	0.233	0.810	1.000
Normal vs. AIP					
Blood urea nitrogen	0.940	**0.004**	**0.007**	0.857	0.950
eGFR (mL/min/1.73 m^2^)	0.906	**0.001**	**0.007**	0.762	0.950
AST	0.849	**0.013**	**0.016**	0.810	0.850
ALT	0.838	**0.028**	**0.029**	0.762	0.900
Citrulline	0.855	**0.003**	**0.007**	0.818	0.900
Ornithine	0.725	**0.029**	**0.029**	0.857	0.550
Tyrosine	0.855	**0.002**	**0.007**	0.952	0.750
Valine	0.818	**0.003**	**0.007**	0.810	0.750
Sarcosine	0.875	**0.004**	**0.007**	0.762	0.900
Phosphatidylcholines diacyl C32:1	0.831	**0.028**	**0.029**	0.905	0.750
Phosphatidylcholines diacyl C36:1	0.833	**0.001**	**0.007**	0.619	1.000
Phosphatidylcholines diacyl C36:3	0.863	**0.004**	**0.007**	1.000	0.650
SM C16:0	0.777	**0.007**	**0.009**	0.714	0.750
SM C24:0	0.786	**0.005**	**0.007**	0.667	0.900
SM C24:1	0.788	**0.004**	**0.007**	0.476	1.000

AST—aspartate aminotransferase; ALT—alanine aminotransferase; AUC—area under the curve; AIP—acute intermittent porphyria; eGFR—estimated glomerular filtration rate; FDR—false discovery rate. Numbers in **bold** indicate statistical significance (*p* < 0.05).

## Data Availability

The data used to support the findings of this study are included in the article.

## Supplementary Materials

The following are available online at https://www.mdpi.com/article/10.3390/cells10102579/s1, Table S1: Disease characteristics for asymptomatic carriers and AIP patients, Table S2: Distribution of 144 metabolites between normal, asymptomatic carriers, and AIP patients.
