# Supplementary material for: Profiling of Serum Metabolites of Acute Intermittent Porphyria and Asymptomatic HMBS Mutation Carriers
Source: Cells. 2021 Sep 28;10(10):2579. doi: 10.3390/cells10102579 (PMC8533736; doi:10.3390/cells10102579)
Supplement: Supplementary file 1 [file cells-10-02579-s001.zip › cells-1368420-supplementary.pdf]

Table S1. Disease characteristics for asymptomatic carriers and AIP patients.

	**Carrier**	**AIP**	***p*-value**
	N=5	N=21	
Clinical features at diagnosis			
GI symptoms[c]		21 (100%)	
CNS[d]		5 (24%)	
PNS[e]		4 (19%)	
Chronic sequel		1 (4.7%), mild motor paresis	
Urine-PBG (mg/24hrs)			0.91[a]
No attack	30.9 (3.1, 43.2)	23.2 (10.4, 26.3)	
Attack	-	92.5 (76.5, 123.4)	-
Urine-ALA (mg/24hrs)			0.36[a]
No attack	7.4 (4.4, 20.5)	5.5 (3.5, 7.9)	
Attack	-	33.5 (20.0, 57.3)	-
HMBS gene mutation			0.75[b]
c.77G>A	2 (40%)	5 (23.8%)	
c.652G>A	0	2 (9.5%)	
Others	3 (60%)	14 (66.7%)	
Frequency of attack at early stage [f]	-		-
≤1/year		7 (33.33%)	
1-3/year		3 (14.28%)	
3-6/year		4 (19.05%)	
>6/year		7 (33.33%)	
Frequency of attack within recent two years [g]	-		-
No attacks		13 (61.9%)	
≤1/year		4 (19.05%)	
1-3/year		1 (4.76%)	
3-6/year		2 (9.53%)	
>6/year		1 (4.76%)	

GI, gastrointestinal; CNS, central nervous system; PNS, peripheral nervous system; PBG, porphobilinogen; ALA, δ-aminolevulinic acid.Data was presented as median and interquartile for urine PBG and ALA; n(%) for HMBS gene mutation and frequency of attack.There were 5 missing values in urine-PBG no attack (carrier=3; AIP=2); 6 missing values in urine-PBG attack (AIP=6); 4 missing values in urine-ALA no attack (carrier=2; AIP=2); 6 missing values in urine-ALA attack (AIP=6).

[a], Mann-Whitney U test; [b], Fisher's exact test; [c], abdominal pain, nausea, vomitus or constipation; [d], convulsion, conscious disturbance, severe and new onset headache before or at a diagnosis of AIP; [e], clinical and/or eletrophysiologic events of peripheral neuropathies including acute painful polyneuropathy, subacute sensorimotor polyneuropathy, acute motor axonal neuropathy or drop wrists with bilateral radial motor neuropathy before or at a diagnosis of AIP; [f]. observed within one to two years at disease onset; [g], frequency of attacks after Heme arginate for acute attack and/or prophylactic Heme arginate for 5 patients or givosiran for 2 patientsTable S2. Distribution of 144 metabolites between normal, asymptomatic carriers, and AIP patients.

Table S2. Distribution of 144 350 metabolites between normal, asymptomatic carriers, and AIP patients.

	**Normal**	**Asymptomatic Carrier**	**AIP**	***p*-value**
(μM)	N=20	N=5	N=21	
C0	44.65 (36.40, 49.30)	40.50 (40.00, 43.00)	47.50 (32.90, 51.90)	0.93
C2	6.74 (5.42, 7.30)	7.22 (7.22, 9.44)	8.65 (6.51, 13.00)[‡]	0.02*
C3	0.33 (0.25, 0.40)	0.38 (0.27, 0.45)	0.37 (0.31, 0.44)	0.48
C3-DC (C4-OH)	0.03 (0.03, 0.04)	0.03 (0.03, 0.04)	0.05 (0.04, 0.05)[‡]	0.0003*
C4	0.19 (0.16, 0.21)	0.19 (0.16, 0.20)	0.27 (0.20, 0.33)	0.008*
C5	0.11 (0.09, 0.14)	0.11 (0.09, 0.11)	0.14 (0.09, 0.16)	0.39
C5-DC (C6-OH)	0.02 (0.01, 0.02)	0.02 (0.01, 0.02)	0.02 (0.02, 0.03)[‡§]	<0.0001*
C5-OH (C3-DC-1)	0.02 (0.02, 0.03)	0.02 (0.02, 0.03)	0.03 (0.03, 0.04)[‡]	0.001*
C7-DC	0.03 (0.02, 0.04)	0.03 (0.03, 0.05)	0.04 (0.03, 0.06)[‡]	0.02*
C8	0.19 (0.15, 0.22)	0.19 (0.14, 0.23)	0.23 (0.19, 0.28)	0.16
C9	0.02 (0.02, 0.03)	0.03 (0.02, 0.03)	0.03 (0.02, 0.04)	0.02*
C10	0.22 (0.17, 0.26)	0.24 (0.17, 0.27)	0.26 (0.22, 0.39)	0.13
C12	0.07 (0.07, 0.09)	0.08 (0.06, 0.08)	0.10 (0.07, 0.12)	0.08
C14:1	0.09 (0.07, 0.10)	0.08 (0.08, 0.09)	0.09 (0.09, 0.12)	0.05
C14:2	0.05 (0.03, 0.06)	0.04 (0.04, 0.05)	0.06 (0.05, 0.07)	0.08
C14:2-OH	0.007 (0.007, 0.009)	0.006 (0.005, 0.007)	0.01 (0.007, 0.01)	0.06
C16	0.13 (0.11, 0.16)	0.13 (0.12, 0.14)	0.13 (0.11, 0.16)	0.91
C16:1	0.03 (0.02, 0.04)	0.03 (0.03, 0.03)	0.04 (0.03, 0.04)	0.10
C18	0.04 (0.03, 0.05)	0.04 (0.04, 0.04)	0.04 (0.03, 0.04)	0.96
C18:1	0.11 (0.09, 0.15)	0.12 (0.12, 0.13)	0.13 (0.11, 0.15)	0.73
C18:2	0.06 (0.04, 0.09)	0.07 (0.06, 0.09)	0.07 (0.05, 0.09)	0.54
Alanine	312.50 (241.50, 367.00)	239.00 (231.00, 254.00)	285.00 (223.00, 322.00)	0.34
Arginine	71.90 (51.90, 86.85)	63.40 (48.00, 69.30)	68.00 (48.90, 85.10)	0.56
Asparagine	41.55 (38.90, 46.85)	41.30 (35.70, 41.80)	41.30 (37.50, 44.20)	0.50
Aspartate	3.90 (3.15, 5.00)	4.90 (4.50, 5.70)	5.70 (4.60, 7.70)[‡]	0.01*
Citrulline	23.85 (20.85, 28.10)	22.80 (21.00, 24.40)	34.30 (31.30, 46.70)[‡§]	0.0001*
Glutamine	546.50 (499.00, 605.50)	536.00 (521.00, 593.00)	534.00 (486.00, 593.00)	0.95
Glutamate	35.00 (28.55, 40.85)	41.10 (32.90, 41.90)	32.80 (26.40, 55.90)	0.89
Glycine	205.00 (177.00, 238.00)	184.00 (180.00, 191.00)	244.00 (215.00, 263.00)	0.03*
Histidine	77.55 (72.10, 82.25)	74.30 (70.80, 79.50)	81.00 (73.60, 91.80)	0.23
Isoleucine	70.05 (63.75, 76.55)	64.50 (51.40, 66.20)	56.60 (51.40, 64.70)[‡]	0.002*
Leucine	116.50 (104.50, 134.50)	111.00 (91.70, 127.00)	102.00 (86.50, 112.00)[‡]	0.03*
Lysine	179.00 (160.00, 203.00)	180.00 (156.00, 195.00)	170.00 (144.00, 197.00)	0.57
Methionine	24.10 (22.80, 25.80)	21.30 (21.10, 23.30)	22.20 (19.40, 25.00)	0.07
Ornithine	60.25 (57.30, 83.45)	56.10 (50.10, 66.60)	82.00 (67.40, 101.00)[‡§]	0.009*
Phenylalanine	63.85 (60.70, 68.55)	63.10 (56.80, 64.90)	59.60 (51.30, 63.60)	0.07
Proline	143.00 (120.00, 150.50)	143.00 (142.00, 173.00)	153.00 (136.00, 161.00)	0.23
Serine	119.00 (97.40, 129.00)	122.00 (119.00, 125.00)	100.00 (94.40, 118.00)	0.14
Threonine	111.50 (107.00, 125.00)	101.00 (85.40, 108.00)	122.00 (96.60, 136.00)	0.33
Tryptophan	66.00 (58.60, 68.25)	57.20 (53.50, 59.20)	54.70 (44.90, 58.30)[‡]	0.02*
Tyrosine	67.05 (61.70, 70.50)	59.10 (51.90, 62.30)	54.40 (44.60, 58.30)[‡]	0.0002*
Valine	235.50 (214.00, 274.00)	227.00 (160.00, 238.00)	186.00 (166.00, 208.00)[‡]	0.003*
ADMA	0.40 (0.35, 0.50)	0.40 (0.30, 0.40)	0.50 (0.40, 0.60)	0.02*
Kynurenine	1.75 (1.60, 2.10)	1.70 (1.60, 1.70)	2.70 (2.50, 3.50)[‡§]	<0.0001*
Sarcosine	3.85 (3.50, 4.50)	5.00 (3.50, 5.00)	5.60 (5.10, 8.60)[‡]	0.0001*
SDMA	0.40 (0.40, 0.50)	0.40 (0.40, 0.40)	0.80 (0.50, 0.90)[‡§]	<0.0001*
t4-OH-Pro	10.75 (8.80, 13.60)	13.60 (10.80, 16.40)	16.40 (11.60, 18.00)	0.06
Taurine	88.15 (73.65, 97.95)	88.00 (69.60, 116.00)	79.50 (59.70, 110.00)	0.59
lysoPC a C16:0	69.40 (59.80, 74.60)	61.10 (57.70, 61.50)	68.20 (62.60, 90.80)	0.08
lysoPC a C16:1	1.64 (1.39, 1.93)	1.44 (1.29, 1.64)	2.05 (1.71, 2.62)	0.007*
lysoPC a C17:0	1.02 (0.83, 1.24)	0.80 (0.78, 1.10)	1.12 (0.97, 1.52)	0.03*

lysoPC a C18:0	17.95 (16.40, 21.20)	16.20 (14.10, 18.20)	21.00 (17.70, 29.60)	0.03*
lysoPC a C18:1	10.80 (8.78, 12.35)	10.30 (10.00, 10.40)	12.40 (11.00, 16.50)	0.01*
lysoPC a C18:2	23.45 (17.90, 27.75)	20.90 (18.30, 22.60)	24.50 (19.30, 28.70)	0.31
lysoPC a C20:3	1.08 (0.96, 1.34)	0.99 (0.85, 1.13)	1.06 (0.95, 1.45)	0.69
lysoPC a C20:4	4.02 (3.56, 5.05)	4.36 (4.04, 4.66)	3.81 (3.21, 4.26)	0.48
lysoPC a C24:0	0.13 (0.12, 0.15)	0.12 (0.12, 0.13)	0.16 (0.14, 0.17)‡§	0.006*
lysoPC a C26:0	0.13 (0.09, 0.15)	0.12 (0.08, 0.12)	0.13 (0.09, 0.14)	0.68
lysoPC a C26:1	0.04 (0.03, 0.04)	0.03 (0.03, 0.04)	0.05 (0.04, 0.05)	0.05
lysoPC a C28:0	0.12 (0.09, 0.13)	0.10 (0.09, 0.12)	0.13 (0.11, 0.16)	0.51
lysoPC a C28:1	0.13 (0.11, 0.15)	0.12 (0.09, 0.14)	0.15 (0.14, 0.19)	0.01*
PC aa C24:0	0.06 (0.04, 0.07)	0.05 (0.04, 0.05)	0.05 (0.04, 0.07)	0.53
PC aa C28:1	1.21 (1.04, 1.35)	1.65 (1.64, 2.08)	1.92 (1.52, 2.16)‡	0.001*
PC aa C30:0	1.51 (1.26, 1.80)	2.26 (2.11, 2.40)	2.40 (1.87, 3.39)‡	0.0004*
PC aa C32:0	10.45 (8.58, 11.70)	10.80 (9.80, 11.90)	12.80 (11.40, 14.50)‡	0.009*
PC aa C32:1	3.78 (2.70, 5.42)	5.48 (4.80, 5.56)	6.57 (5.28, 10.70)‡	0.001*
PC aa C32:2	1.50 (1.06, 2.05)	1.60 (1.44, 2.09)	2.27 (1.84, 3.57)‡	0.01*
PC aa C32:3	0.19 (0.18, 0.26)	0.22 (0.21, 0.22)	0.27 (0.23, 0.29)‡	0.003*
PC aa C34:1	114.00 (103.00, 128.00)	119.00 (118.00, 119.00)	126.00 (119.00, 138.00)	0.08
PC aa C34:2	225.50 (131.50, 361.00)	138.00 (136.00, 142.00)	140.00 (130.00, 176.00)	0.81
PC aa C34:3	5.88 (5.05, 7.17)	5.45 (5.42, 5.69)	8.42 (6.52, 9.69)	0.005*
PC aa C34:4	0.58 (0.46, 0.85)	0.55 (0.53, 0.66)	0.66 (0.55, 0.99)	0.34
PC aa C36:0	4.87 (2.47, 6.52)	3.17 (2.93, 3.92)	3.06 (2.29, 4.59)	0.47
PC aa C36:1	22.65 (17.70, 28.75)	29.60 (26.20, 29.90)	41.20 (29.30, 44.00)‡	0.0007*
PC aa C36:2	131.00 (116.50, 182.50)	122.00 (122.00, 128.00)	127.00 (123.00, 155.00)	0.68
PC aa C36:3	59.30 (49.80, 64.05)	65.50 (59.30, 73.70)	77.80 (65.40, 87.60)‡	0.0003*
PC aa C36:4	111.00 (99.50, 136.50)	117.00 (108.00, 123.00)	117.00 (105.00, 130.00)	0.88
PC aa C36:5	5.60 (4.54, 7.44)	6.34 (5.55, 10.40)	7.96 (5.61, 9.10)	0.12
PC aa C36:6	0.28 (0.18, 0.32)	0.26 (0.24, 0.30)	0.33 (0.27, 0.39)	0.12
PC aa C38:0	3.13 (2.71, 4.07)	3.00 (2.76, 3.28)	3.72 (3.05, 4.16)	0.40
PC aa C38:3	26.25 (20.95, 31.45)	28.10 (25.00, 30.00)	29.60 (24.80, 35.70)	0.21
PC aa C38:4	67.30 (53.50, 81.45)	75.10 (66.40, 76.20)	73.50 (60.40, 82.50)	0.45
PC aa C38:5	19.05 (15.85, 22.90)	20.60 (20.10, 25.00)	26.00 (22.00, 29.60)‡	0.02*
PC aa C38:6	58.20 (53.15, 67.95)	54.70 (50.10, 56.90)	66.60 (57.10, 75.70)	0.34
PC aa C40:1	0.42 (0.39, 0.51)	0.44 (0.39, 0.48)	0.46 (0.39, 0.50)	0.98
PC aa C40:2	0.30 (0.20, 0.34)	0.36 (0.31, 0.44)	0.40 (0.30, 0.51)‡	0.03*
PC aa C40:3	0.48 (0.41, 0.57)	0.54 (0.51, 0.55)	0.62 (0.55, 0.74)‡	0.004*
PC aa C40:4	2.33 (1.90, 2.82)	2.45 (2.36, 2.53)	2.95 (2.35, 3.47)‡	0.04*
PC aa C40:5	5.93 (4.44, 6.56)	4.85 (4.52, 5.94)	6.38 (5.14, 8.29)	0.09
PC aa C40:6	18.90 (16.00, 24.90)	17.50 (14.70, 22.30)	24.30 (20.00, 28.20)	0.11
PC aa C42:0	0.56 (0.50, 0.77)	0.67 (0.65, 0.86)	0.68 (0.52, 0.88)	0.48
PC aa C42:1	0.40 (0.30, 0.50)	0.38 (0.36, 0.50)	0.40 (0.31, 0.47)	0.94
PC aa C42:2	0.29 (0.24, 0.35)	0.34 (0.29, 0.34)	0.34 (0.28, 0.39)	0.45
PC aa C42:4	0.17 (0.15, 0.20)	0.19 (0.19, 0.22)	0.22 (0.18, 0.26)‡	0.009*
PC aa C42:5	0.25 (0.20, 0.28)	0.27 (0.26, 0.29)	0.31 (0.27, 0.42)‡	0.003*
PC aa C42:6	0.25 (0.17, 0.28)	0.34 (0.29, 0.36)	0.39 (0.27, 0.51)‡	0.01*
PC ae C30:0	0.17 (0.15, 0.20)	0.25 (0.22, 0.29)	0.22 (0.20, 0.28)	0.003*
PC ae C30:2	0.05 (0.05, 0.06)	0.06 (0.05, 0.06)	0.06 (0.06, 0.07)‡	0.006*
PC ae C32:1	1.93 (1.74, 2.37)	2.18 (1.97, 2.35)	2.24 (2.07, 2.77)	0.25
PC ae C32:2	0.44 (0.35, 0.54)	0.57 (0.51, 0.58)	0.60 (0.53, 0.65)‡	0.008*
PC ae C34:0	0.71 (0.58, 0.97)	0.75 (0.71, 0.91)	0.91 (0.78, 1.10)	0.13
PC ae C34:1	5.50 (5.01, 6.83)	6.03 (5.48, 6.24)	7.09 (6.33, 8.21)‡	0.03*
PC ae C34:2	9.10 (8.12, 10.90)	10.30 (9.98, 10.70)	10.80 (9.38, 12.40)	0.11
PC ae C34:3	7.14 (5.95, 8.31)	7.89 (7.41, 8.10)	8.16 (6.85, 10.30)	0.16
PC ae C36:0	0.55 (0.45, 0.71)	0.60 (0.54, 0.62)	0.63 (0.58, 0.80)	0.26
PC ae C36:1	12.60 (9.96, 16.35)	12.80 (10.50, 12.90)	14.20 (11.10, 17.20)	0.22
PC ae C36:2	9.61 (7.38, 11.35)	9.74 (7.90, 9.99)	11.70 (9.65, 12.80)	0.02*
PC ae C36:3	5.34 (4.68, 6.42)	5.92 (5.69, 6.09)	6.50 (5.31, 7.18)	0.12

PC ae C36:4	12.45 (11.30, 14.45)	16.50 (11.90, 17.60)	13.50 (12.10, 15.10)	0.36
PC ae C36:5	8.45 (7.54, 10.07)	9.85 (6.95, 11.00)	8.75 (7.82, 11.00)	0.86
PC ae C38:0	0.92 (0.68, 1.43)	0.85 (0.74, 0.99)	1.03 (0.87, 1.34)	0.52
PC ae C38:3	4.17 (3.08, 4.83)	3.89 (3.78, 4.62)	4.60 (3.68, 4.82)	0.47
PC ae C38:4	6.93 (6.10, 8.34)	8.51 (6.79, 9.65)	7.54 (6.52, 8.96)	0.52
PC ae C38:5	10.20 (9.61, 12.60)	13.00 (9.75, 13.20)	12.20 (10.70, 12.70)	0.38
PC ae C38:6	3.95 (3.37, 4.57)	5.02 (4.61, 5.14)	5.10 (4.18, 5.28)‡	0.03*
PC ae C40:1	1.21 (0.88, 1.79)	1.02 (0.93, 1.11)	1.25 (1.16, 1.48)	0.27
PC ae C40:2	1.19 (1.07, 1.33)	1.29 (1.24, 1.84)	1.52 (1.29, 1.91)‡	0.005*
PC ae C40:3	1.32 (1.22, 1.56)	1.52 (1.47, 1.53)	1.52 (1.35, 1.68)	0.22
PC ae C40:4	1.47 (1.35, 1.78)	1.96 (1.89, 2.03)	1.73 (1.52, 1.88)	0.19
PC ae C40:5	2.82 (2.35, 3.25)	3.26 (2.96, 3.49)	3.34 (2.80, 3.58)	0.11
PC ae C40:6	3.25 (2.99, 4.19)	3.42 (3.27, 3.45)	3.92 (3.30, 4.30)	0.29
PC ae C42:1	0.43 (0.29, 0.58)	0.36 (0.34, 0.37)	0.45 (0.35, 0.52)	0.41
PC ae C42:2	0.44 (0.36, 0.57)	0.43 (0.38, 0.44)	0.52 (0.46, 0.58)	0.19
PC ae C42:3	0.88 (0.69, 1.08)	0.87 (0.83, 0.90)	0.85 (0.80, 1.07)	0.66
PC ae C42:4	0.78 (0.72, 0.97)	1.00 (0.99, 1.06)	0.92 (0.79, 1.07)	0.20
PC ae C42:5	1.35 (1.17, 1.66)	2.04 (1.92, 2.06)	1.69 (1.49, 2.09)	0.12
PC ae C44:3	0.12 (0.10, 0.15)	0.13 (0.11, 0.14)	0.15 (0.13, 0.17)	0.15
PC ae C44:4	0.32 (0.28, 0.42)	0.42 (0.40, 0.43)	0.36 (0.31, 0.44)	0.41
PC ae C44:5	0.86 (0.69, 1.21)	1.59 (1.34, 1.67)	1.22 (0.93, 1.61)	0.09
PC ae C44:6	0.95 (0.76, 1.29)	1.49 (1.39, 1.78)	1.18 (0.89, 1.61)	0.19
SM (OH) C14:1	5.11 (4.32, 5.71)	4.27 (4.21, 6.67)	5.59 (4.99, 6.75)	0.08
SM (OH) C16:1	3.05 (2.71, 3.45)	3.12 (2.56, 4.24)	3.38 (2.99, 3.82)	0.11
SM (OH) C22:1	13.10 (11.70, 14.40)	15.20 (13.40, 16.00)	15.30 (13.70, 16.90)	0.10
SM (OH) C22:2	10.35 (9.70, 11.90)	10.60 (10.60, 13.00)	11.70 (10.20, 13.70)	0.18
SM (OH) C24:1	1.16 (1.03, 1.36)	1.57 (1.41, 1.82)	1.57 (1.33, 1.79)‡	0.008*
SM C16:0	118.50 (102.50, 131.00)	127.00 (127.00, 128.00)	142.00 (127.00, 150.00)‡	0.009*
SM C16:1	17.85 (16.80, 20.30)	19.90 (17.70, 20.90)	21.40 (17.90, 24.30)	0.08
SM C18:0	22.05 (19.35, 24.10)	24.80 (21.60, 25.70)	23.60 (21.40, 26.40)	0.23
SM C18:1	13.40 (11.50, 14.90)	14.70 (12.20, 16.40)	13.50 (11.50, 15.30)	0.68
SM C20:2	0.48 (0.40, 0.56)	0.54 (0.53, 0.54)	0.58 (0.53, 0.62)‡	0.04*
SM C24:0	17.80 (16.00, 23.90)	23.40 (22.70, 27.20)	26.90 (22.10, 29.00)‡	0.008*
SM C24:1	55.75 (47.70, 61.85)	60.70 (57.80, 62.60)	67.80 (59.60, 74.90)‡	0.009*
SM C26:0	0.19 (0.16, 0.20)	0.22 (0.18, 0.23)	0.23 (0.20, 0.27)‡	0.006*
SM C26:1	0.34 (0.30, 0.44)	0.47 (0.44, 0.52)	0.48 (0.40, 0.69)‡	0.007*

Data was presented as median and interquartile.

*p<0.05 by Kruskal-Wallis test.

‡ p<0.05 for Dunn's multiple comparison test between normal and AIP group.

§ p<0.05 for Dunn's multiple comparison test between carrier and AIP group.
